# Design of Vif-Derived Peptide Inhibitors with Anti-HIV-1 Activity by Interrupting Vif-CBFβ Interaction

**DOI:** 10.3390/v16040490

**Published:** 2024-03-22

**Authors:** Yanxin Gai, Sizhu Duan, Shiqi Wang, Kaifeng Liu, Xin Yu, Chumeng Yang, Guoqing Li, Yan Zhou, Bin Yu, Jiaxin Wu, Chu Wang, Xianghui Yu

**Affiliations:** 1 National Engineering Laboratory for AIDS Vaccine, School of Life Sciences, Jilin University, Changchun 130012, China; gaiyx20@mails.jlu.edu.cn (Y.G.); duansz16@mails.jlu.edu.cn (S.D.); wangsq17@mails.jlu.edu.cn (S.W.); 52261300050@stu.ecnu.edu.cn (X.Y.); yangcm1319@mails.jlu.edu.cn (C.Y.); ligq21@mails.jlu.edu.cn (G.L.); julysec@jlu.edu.cn (Y.Z.); yubin@jlu.edu.cn (B.Y.); wujiaxin@jlu.edu.cn (J.W.); 2 Key Laboratory for Molecular Enzymology and Engineering, The Ministry of Education, School of Life Sciences, Jilin University, Changchun 130012, China; liukf1220@mails.jlu.edu.cn

**Keywords:** peptide inhibitor, HIV-1, Vif, CBFβ, APOBEC3G

## Abstract

One of the major functions of the accessory protein Vif of human immunodeficiency virus type 1 (HIV-1) is to induce the degradation of APOBEC3 (A3) family proteins by recruiting a Cullin5-ElonginB/C-CBFβ E3 ubiquitin ligase complex to facilitate viral replication. Therefore, the interactions between Vif and the E3 complex proteins are promising targets for the development of novel anti-HIV-1 drugs. Here, peptides are designed for the Vif-CBFβ interaction based on the sequences of Vif mutants with higher affinity for CBFβ screened by a yeast surface display platform. We identified two peptides, VMP-63 and VMP-108, that could reduce the infectivity of HIV-1 produced from A3G-positive cells with IC50 values of 49.4 μM and 55.1 μM, respectively. They protected intracellular A3G from Vif-mediated degradation in HEK293T cells, consequently increasing A3G encapsulation into the progeny virions. The peptides could rapidly enter cells after addition to HEK293T cells and competitively inhibit the binding of Vif to CBFβ. Homology modeling analysis demonstrated the binding advantages of VMP-63 and VMP-108 with CBFβ over their corresponding wild-type peptides. However, only VMP-108 effectively restricted long-term HIV-1 replication and protected A3 functions in non-permissive T lymphocytes. Our findings suggest that competitive Vif-derived peptides targeting the Vif-CBFβ interaction are promising for the development of novel therapeutic strategies for acquired immune deficiency syndrome.

## 1. Introduction

The majority of the replication steps of the human immunodeficiency virus (HIV) are influenced by interacting with host proteins [1,2,3]. Human apolipoprotein B mRNA-editing catalytic polypeptide-like 3 (APOBEC3; A3) enzymes are initially discovered as host restriction factors that inhibit the replication of HIV-1 [4,5,6], especially APOBEC3G (A3G), which shows the most potent inhibitory activity among the A3 family members [7]. A3s can be packaged in budding HIV-1 particles after binding to the viral RNA and trigger a high frequency of mutations in the viral genome by deaminating cytidines to uridines during single-stranded minus-strand DNA synthesis [8,9,10,11]. A3G prefers to recognize 5′-CC-3′ dinucleotide sites, resulting in GG-to-AG mutations [9], while A3F, A3D, and A3H prefer 5′-TC-3′ sites, resulting in GA-to-AA mutations [12,13,14]. As counter mechanisms, HIV-1 accessory protein Vif mediates the degradation of A3 proteins by recruiting an E3 ubiquitin ligase complex consisting of the scaffold protein Cullin5 (CUL5), adaptor proteins ElonginB (EloB) and ElonginC (EloC), catalytic unit RBX1, and chaperone core-binding factor beta (CBFβ) [4,15,16,17,18]. It has also been reported that Vif binds to the mRNA of A3G, down-regulating its expression and stability to directly inhibit the deaminase activity of A3G [19,20,21].

Targeting the protein–protein interactions (PPIs) between Vif and the proteins in the E3 ubiquitin ligase complex to restore the mutagenic activity of A3 enzymes is an important direction for the development of anti-HIV-1 drugs [22]. Extensive studies have been conducted to develop inhibitors to disrupt the interactions, and most of them target the Vif-A3G and Vif-EloC interfaces [23,24,25,26,27,28,29,30,31]. The Vif-CBFβ interaction is also a promising target because the absence of CBFβ blocks the assembly of the E3 complex hijacked by Vif, thus protecting the A3 proteins from degradation [17,32,33]. CBFβ is a cofactor of the transcription factor RUNX protein family, which regulates the expression of a range of genes associated with hematopoietic and bone cell development [34]. A study showed that the binding of RUNX1 to CBFβ regulates the expression of A3 genes, allowing them to exert the antiviral effect [35]. Nevertheless, Vif competitively binds to CBFβ and inhibits it from forming heterodimers with RUNX1, antagonizing the upregulation of A3G protein expression [35,36].

Several features were discovered in the interaction between Vif and CBFβ in the crystal structure of a Vif-CBFβ-CUL5-EloB-EloC-RBX1 complex (PDB: 4N9F) [37]: (i) a larger binding interface between the hydrophobic surface of Vif with CBFβ than with other complex members, leaving a positively charged surface exposed, which contributes to a higher affinity of Vif to CBFβ than RUNX1; (ii) the N-terminal (residues 6 to 12) of Vif forms an antiparallel β-sheet with the β-strand S3 (residues 65 to 70) from CBFβ to maintain a stable and functional Vif-CBFβ complex; and (iii) the C-terminal of CBFβ (residues 149 to 155) binds to the pocket formed by two regions (residues 100 to 108 and 118 to 126) from Vif, further stabilizing the conformation of Vif α-region. In addition to the binding information directly reflected by the crystal structure, many studies also investigated the interaction sites between Vif and CBFβ. Sites such as ^5^WQVMIVW^11^, W21, W38, L64, I66, ^84^GXSIEW^89^, ^102^LADQLIH^108^, F112, and F115 of Vif exhibit important roles in binding with CBFβ [36,38,39,40,41,42,43]. From the perspective of CBFβ, the binding to Vif involves the region from residues 15 to 126 [36,37,38].

We recently identified a novel small-molecule compound CV-3 and a dominant-negative mutant Vif-6M that could both restrict HIV-1 efficiently by targeting the Vif-CBFβ interface [44,45]. It has been found that PPIs with a buried surface area smaller than 2000 Å^2^ are easily inhibited by small molecules, while PPIs with larger areas are generally inhibited easily by peptides [46]. Compared with small-molecule inhibitors, peptides are well tolerated, safer, more specific, and more suitable to act on the large interface between Vif and CBFβ (>4000 Å^2^) [37]. In previous studies, Vif-derived peptide antagonists targeting Vif dimerization and Vif-A3G interaction have also been proven effective [47,48,49,50]. Therefore, in this study, we aim to design peptide inhibitors targeting the Vif-CBFβ interaction based on the sequences of dominant-negative Vif mutants, which can be screened by the platform we established, to restrict HIV-1 replication.

Here, we identify two efficient peptides towards this target, VMP-63 and VMP-108 (IC_50_ = 49.4 μM and 55.1 μM, respectively), which significantly restrict the infection of HIV-1 without compromising cell viability. VMP-63 and VMP-108 were shown to protect A3G from Vif-mediated degradation by blocking the interaction between Vif and CBFβ, thereby enhancing A3G incorporation into HIV-1 virions to reduce viral infectivity. These two peptides are potential candidates for further optimization and development of anti-HIV-1 drugs.

## 2. Materials and Methods

### 2.1. Cell Lines, Culture, and Transfection

HEK293T cells (cat#CRL-11268) were purchased from the American Type Culture Collection. TZM-bl, CEM, and CEM-SS cells were obtained from the National Institutes of Health, HIV Reagent Program (NIH-HRP). The chronically infected cell lines H9/HXB2Neo and Jurkat/HXB2Neo were constructed as previously described [51]. HEK293T and TZM-bl cells were cultured in Dulbecco’s Modified Eagle Medium (DMEM) containing 10% fetal bovine serum (FBS) at 37 °C in 5% CO_2_. CEM, CEM-SS, H9/HXB2Neo, and Jurkat/HXB2Neo cells were cultured in Roswell Park Memorial Institute (RPMI) 1640 medium with 10% FBS. Following the manufacturer’s protocol, cells were transfected using jetPRIME (Polyplus-transfection, Illkirch, France).

### 2.2. Plasmids, Antibodies, and Protein Expression

The full-length infectious molecular clone of HIV-1 strain NL4-3 (pNL4-3) was obtained from NIH-HRP. The Vif-defective variant pNL4-3ΔVif and plasmids pVR1012-A3G-cmyc, pVR1012-Vif-HA, pVR1012-CUL5-cmyc, pVR1012-CBFβ-cmyc, pVR1012-EloB-cmyc, pVR1012-EloC-cmyc, pVR1012-Vif-YFP, and pCBFβ-CFP were constructed previously [15,44]. The antibodies used are as follows: anti-HA antibody and anti-GAPDH antibody (Covance, Emeryville, CA, USA), anti-cmyc antibody (Millipore, Billerica, MA, USA), anti-Vif antibody (Invitrogen, Carlsbad, CA, USA), rabbit monoclonal antibody to CBFβ, and goat anti-rabbit IgG conjugated to FITC (Abcam, Cambridge, MA, USA). Pr55 Gag and capsid p24 were detected with a monoclonal anti-HIV-1 capsid antibody generated by an HIV-1 p24 hybridoma (NIH-HRP). The CBFβ protein was expressed in Escherichia coli strain BL21 and purified using Ni NTA-His affinity columns as previously described [44]. The purified protein was stored at −80 °C.

### 2.3. Yeast Surface Display

The Vif mutant libraries with mutation frequencies of 3.5‰, 4.6‰, and 5.8‰ were constructed using *Saccharomyces cerevisiae* strain EBY100 (Invitrogen), according to the protocol of a Diversify PCR Random Mutagenesis Kit (Clontech, Mountain View, CA, USA) [45]. The plasmid pool capacity of the three mutant libraries was above 2 × 10^5^ (3.5‰: 2.8 × 10^5^; 4.6‰: 2.5 × 10^5^; and 5.8‰: 2 × 10^5^). Yeast cells expressing different proteins were cultured to a certain cell density and induced to express the Vif mutants, followed by incubation with the CBFβ protein. After successive incubation with the CBFβ monoclonal antibody and FITC-labeled goat anti-rabbit IgG antibody, the CBFβ-positive cell population with higher FITC intensity was sorted by flow cytometry (Beckman Coulter, Brea, CA, USA).

### 2.4. Single Sequence Amplification and Peptide Synthesis

The top 0.01% of single yeast cells from the positive cell population were collected directly into 96-well PCR plates pre-dosed with 10 μL of lysis buffer. Subsequently, the Vif mutant fragments were amplified by two rounds of nested PCR for sequencing. The peptides (9–17 mer) were designed according to the high-frequency amino acid mutations and the functional regions of Vif and then synthesized with modifications of N-terminal acetylation and C-terminal amidation (GL Biochem, Shanghai, China). To study the intake rate of peptides into cells, the peptides modified with N-terminal FITC fluorescent were synthesized. A random peptide (DHYRTAGSEFCNILW) was synthesized as a negative control for the fluorescence resonance energy transfer (FRET) assay.

### 2.5. Generation, Purification, and Titration of Virus

The viruses were generated by transfecting the proviral expression plasmids into HEK293T cells. Cell culture supernatants were harvested at 48 to 72 h post-transfection and concentrated through a 20% sucrose cushion by ultracentrifugation at 100,000× *g* for 2 h. The viruses were titrated by an HIV-1 p24 ELISA kit (Xpressbio, Frederick, MD, USA).

### 2.6. Viral Infectivity Assay

The method for detecting viral infectivity has been described previously [45]. Briefly, virus samples were mixed with 1 × 10^4^ TZM-bl cells in 200 µL DMEM containing 10 µg/mL of DEAE-dextran in 96-well plates. After 48 h of incubation, 150 µL of supernatant was removed and 50 µL of firefly luciferase substrate (Promega, Madison, WI, USA) was added into each well to test the relative luciferase units (RLUs), which represent the infection of the virus.

### 2.7. Western Blotting

Cells and viruses were harvested at 48 h after transfection and lysed with loading buffer. The samples were boiled for 10 min at 97 °C, subjected to standard SDS-polyacrylamide gel electrophoresis (SDS-PAGE), and then transferred to nitrocellulose membranes. After incubation with primary and secondary antibodies and a High-sig ECL Western Blotting Substrate (Tanon, Shanghai, China) in order, the membranes were visualized with Tanton-5200 (Tanon). The level of protein was quantified by Image J (LOCI, University of Wisconsin, Madison, WI, USA).

### 2.8. Peptide Intake Assay

HEK293T cells were seeded into 12-well plates one day before peptide incubation, and then 5 μM FITC-labeled peptides were added to the cells at different time points. An equal volume of DMSO was added as a negative control. All samples were collected after the last time point and centrifuged at 3000 rpm for 5 min. After washing three times with PBS, the rate of FITC-positive cells was analyzed by flow cytometry C6 (BD Biosciences, San Jose, CA, USA).

### 2.9. FRET Assay

FRET experiments for detecting protein–protein interaction have been described previously [45]. Briefly, HEK293T cells were transfected with yellow fluorescent protein-fused Vif (Vif-YFP) and cyan fluorescent protein-fused CBFβ (CBFβ-CFP) and cultured for 48 h. FRET interaction between Vif-YFP and CBFβ-CFP was detected using an LS-55 spectrofluorometer (PerkinElmer, Waltham, MA) by following a procedure to remove contributions from the background signal, CFP emission (“bleed-through”), and direct YFP emission (“cross-talk”) from Vif-YFP and CBFβ-CFP emission spectra. Samples were excited at CFP max (440 nm). Fluorescence emission attributable to FRET was then determined by subtracting the normalized emission spectrum of a CBFβ-CFP-only sample and the normalized emission spectrum of a Vif-YFP-only sample from the emission spectrum of the mixed Vif-YFP and CBFβ-CFP samples. The formula for calculating FRET efficiency was described previously [52].

### 2.10. Co-Immunoprecipitation (Co-IP)

Transfected 293T cells were washed with cold PBS, resuspended in 500 μL RIPA Lysis Buffer (Beyotime, Shanghai, China) with cOmplete protease inhibitor cocktail tablets at 4 °C for 45 min, and centrifuged at 12,000 rpm for 15 min. The precleared cell lysates were then mixed with anti-HA antibody-conjugated agarose beads (Roche, Mannheim, Germany) and incubated at 4 °C for 4 h. The samples were washed four times with a washing buffer. The beads were collected by centrifugation and mixed with 30 μL RIPA Lysis Buffer and 10 μL 4× loading buffer. After boiling at 97 °C for 10 min, the samples were separated by SDS-PAGE and detected by immunoblotting.

### 2.11. Molecular Dynamics (MD) Simulations

Systems under study were designated as follows: CBFβ-Vif, CBFβ-VM-63, CBFβ-VM-108, CBFβ-VMP-63M, and CBFβ-VMP-108M. We constructed the 3D structure of CBFβ-Vif and the mutants using Alphafold2 Colab [53,54] based on PDB ID: 4N9F [37]. The peptide system was constructed using local conformation. Our simulation efforts used the AMBER 16′ s pmemd.cuda tool [55], applying the ff14SB [56] and TIP3P [57] force fields via the Leap module for protein and water molecules parameterization, respectively. Solvation of each variant occurred within an octahedral container of TIP3P water, ensuring a 12 Å buffer from the solute’s boundary to the container edges. We enforced PBCs to counteract edge artifacts, alongside ion additions to balance the charge. Hydrogen-containing bonds were restrained with SHAKE [58], and non-bonded electrostatic details were managed using the PME method [59], setting the threshold at 8 Å. Prior to simulation runs, each model underwent an energy minimization to resolve potential steric clashes, applying a combined 2000-step regimen of steepest descent and conjugate gradient techniques. A stepwise heating protocol brought the systems from 0 K to 309.65 K within 50 ps under the NVT conditions. Simulations ran for a 200 ns duration, adhering to the NPT ensemble, with randomized seeding. The constant pressure of 1 bar is maintained by the Berendsen barostat with a pressure-coupling constant of 2 ps, a temperature of 309.65 K, and the Langevin thermostat [60] with a collision frequency of 1 ps. For trajectory examination, we conducted K-means clustering, utilizing the Cpptraj module within Amber16 [61]. The MM-PBSA method facilitated the computation of binding free energies, a crucial determinant in the evaluation of protein–protein affinities [62,63,64].

### 2.12. HIV-1 Replication in T Lymphoblast Cell Lines

A total of 5 × 10^5^ cells (CEM or CEM-SS) were infected with wild-type (WT) or Vif-defective HIV-1 of 5 ng p24 at 37 °C for 4 h. The virus-containing supernatants were then removed, and the cells were washed three times with RPMI 1640 medium. The cells were cultured with peptides for 7 or 11 days. p24 levels were monitored at different time points using the HIV-1 p24 ELISA kit.

### 2.13. Cytotoxicity Assay

The peptides were serially diluted by 2-fold from 100 μM with DMEM and incubated with 2 × 10^4^ HEK293T cells or 1 × 10^4^ CEM or CEM-SS cells in 200 μL DMEM at 37 °C for 48 h. Then, 100 µL of the supernatants were removed, and 100 µL of a CellTiter-Glo^®^ substrate (Promega, Madison, WI, USA) was added into each well to measure the RLU. The RLU reflected the number of viable cells based on the amount of intracellular ATP that reacted with the substrate.

### 2.14. Single Genome Amplification and G-to-A Mutation Analysis

Viral RNA was isolated from the virus-containing supernatants using a TIANamp Virus DNA/RNA Kit (Tiangen Biotech, Beijing, China), according to the manufacturer’s protocol. Then, reverse transcription was performed on the viral RNA using the reverse primer 5R2 (5′-AGATATGTTGTCCTAAGTTATGGAG-3′). The cDNA template was diluted to control the positive rate of PCR products to less than 20%. An 850 bp DNA fragment of the *pol* gene was amplified by nested PCR with a Taq DNA polymerase (Invitrogen) using the outer primers Fwd-1 (5′-GCACTTTAAATTTTCCCATTAGTCC-3′) and Rev-1 (5′-TGCAAAGCTAGATGAATTGCTTGTAAC-3′) and the inner primers Fwd-2 (5′-GTATACTGCATTTACCATACCTAGTATAAAC-3′) and Rev-2 (5′-GGAGGGGTATTGACAAACT-3′) [65]. The PCR products were sequenced to obtain single *pol* gene sequences. Hypermut 2.0, available from https://www.hiv.lanl.gov/content/sequence/HIGHLIGHT/highlighter_top.html (accessed on 30 November 2023), was employed to detect G-to-A and non-G-to-A mutations. All the DNA fragments were compared to the NL4-3 *pol* gene sequence to identify G-to-A mutations and also the nucleotide context of the mutations.

### 2.15. Statistical Analysis

Statistical analysis was performed with GraphPad version 8 (GraphPad Software, Inc., La Jolla, CA, USA). Data were presented as mean ± standard deviation. A *p*-value < 0.05 was considered significant. * indicates *p* < 0.05; **, *p* < 0.01; ***, *p* < 0.001; ns, not significant.

## 3. Results

### 3.1. Design of Peptides Based on the Sequences of Dominant-Negative Vif Mutants

**Figure 1:**
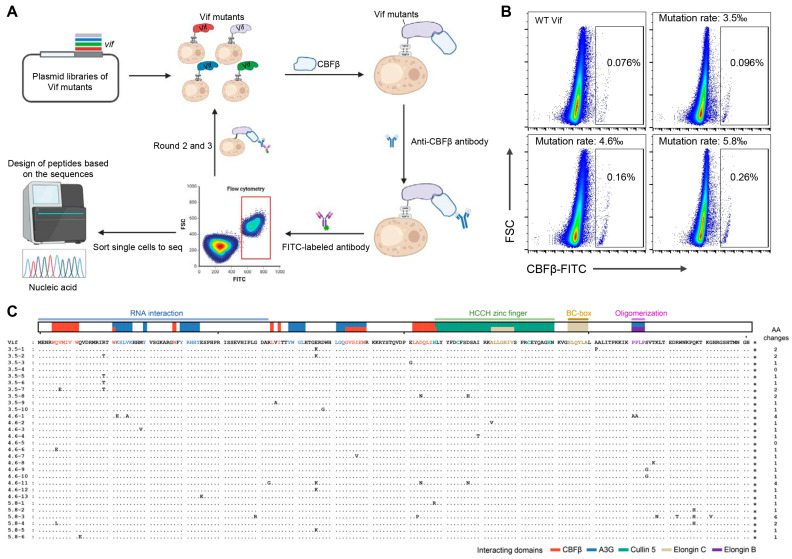
Screening of Vif mutants with higher affinity for CBFβ by yeast surface display. (**A**) Schematic diagram of the screening process. Plasmid libraries with random mutations in the Vif-encoding gene were used for the transduction of yeast cells. The yeast cells displaying Vif mutants were successively incubated with purified CBFβ protein, anti-CBFβ antibody, and FITC-labeled secondary antibody. After three rounds of sorting enrichment by flow cytometry, the top single cells in the FITC-positive cell population were sorted for *vif* fragment amplification and sequencing. (**B**) Fluorescence-activated cell sorting (FACS) plots showing the sorted FITC-positive cells from the third round of enrichment. The FITC-positive rates of cells expressing wild-type (WT) Vif and Vif mutants with the library mutation rate of 3.5‰, 4.6‰, and 5.8‰ are demonstrated in the black rectangular gates, respectively. The top 0.01% of cells from each Vif mutant group were sorted. (**C**) Alignment of the amino acid sequences of sorted Vif mutants. The reported functional domains of Vif are demonstrated in different colors above the sequences [66]. Dots indicate no mutations. A short dash indicates an amino acid deletion. * indicates the stop code. The number of amino acid (AA) changes per sequence is highlighted on the right.

In our previous study, three plasmid libraries with random mutations in the Vif-encoding gene (mutation rate = 3.5‰, 4.6‰, and 5.8‰, respectively) were constructed and used to display Vif mutants on the transduced yeast cells [45]. New Vif mutants with higher affinity for CBFβ than WT Vif were screened herein by three rounds of sorting enrichment (Figure 1A). Comparing the cells expressing WT Vif bound with CBFβ protein (0.076%) as a positive control, 0.096%, 0.16%, and 0.26% FITC-positive cells were detected in the yeast cells transduced with the three Vif mutant libraries, respectively (Figure 1B), indicating a stronger binding capacity of the mutants to CBFβ than that of WT Vif. We selected the top 0.01% of positive cells expressing Vif mutants from each of the three groups for single-cell sorting and sequence amplification. A total of 29 intact Vif mutant sequences were obtained. The alignment showed that the mutations were randomly distributed throughout the sequences (Figure 1C). In the 29 sequences, 29 different mutations were screened out, of which 27 (>90%) were consistent with the mutations shown in our previous data [45].

Peptide candidates were designed based on the amino acid sequences of the sorted Vif mutants. We divided the Vif sequence into several domains according to their reported functions [15,37,39,66,67,68,69,70] (Figure 1C). Some mutations in or beside the known domains or sites of Vif interacting with CBFβ were found in the Vif mutant sequences, such as Q6E/L, V7E, W11G, I87V, A103P, D104N, and H108R [36,39,41,43], which were colored in red in Table 1, confirming that the mutation sites screened by this method were CBFβ high-affinity-specific. Since the N-terminal domain, L64/I66, and the zinc finger structures of Vif are important for its interaction with CBFβ [36,38,39,40,41,42,43], we designed six peptides (9–17 m) stepwise covering most of the mutations occurring in these three regions to analyze their potential to competitively inhibit the Vif-CBFβ interaction (Table 2). Each peptide was designed to introduce 1 to 3 mutation sites. Peptide VMP-6 was designed based on the CBFβ binding region at the N-terminus of Vif, containing the three mutation sites Q6E, Q12E, and R19T. Peptides VMP-60, 63, and 70, which have partially overlapped sequences, were designed based on the key binding sites L64 and I66. VMP-60 contains the mutations G60R, R63G, and E76K, while VMP-63 contains R63G, V65A, and D78G. And VMP-70, which only contains the mutation D78G, was a truncated control of VMP-63. VMP-108 containing the mutations H108R and D117N and VMP-111 containing D117N, I120T, and A123V were both designed for testing the contribution of the zinc finger domain (Table 2).

### 3.2. Peptides VMP-63 and VMP-108 Decrease HIV-1 Infectivity in an A3G-Dependent Manner

**Figure 2:**
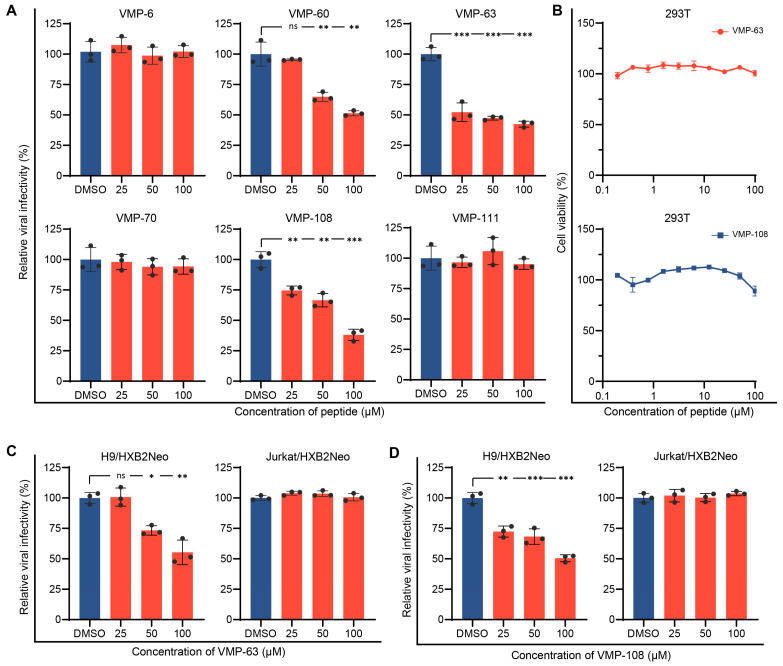
Peptides VMP-63 and VMP-108 decreased HIV-1 infectivity in an A3G-dependent manner. (**A**) The effect of peptide treatment on the infectivity of NL4-3 packaged with A3G. HEK293T cells were co-transfected with pNL4-3 and pVR-A3G-cmyc. Here, 25 μM, 50 μM, or 100 μM of different peptides were added to the cells 4 h post-transfection. DMSO was added as a negative control. The viral infectivity was tested by infecting TZM-bl cells with the culture supernatants of HEK293T cells collected at 48 h post-transfection. The viral infectivity of the DMSO treatment groups was set to 100%. (**B**) Different concentrations of VMP-63 and VMP-108 showed no cytotoxicity in HEK293T cells. The peptides were diluted by 2-fold from 100 μM and incubated with the cells for 48 h. The cell viability of the DMSO-treated group was set to 100%. (**C**,**D**) Chronically infected H9/HXB2Neo cells and Jurkat/HXB2Neo cells were treated with VMP-63 (**C**) or VMP-108 (**D**) at 25 μM, 50 μM, or 100 μM for 48 h. The viral infectivity was tested in TZM-bl cells using the culture supernatants of these two cells. Error bars represent the standard deviation (SD) calculated from three independent experiments. Statistical significance was assessed by the Student’s *t*-test. * indicates *p* < 0.05; **, *p* < 0.01; ***, *p* < 0.001; ns, not significant.

To determine whether the peptides can reduce HIV-1 infectivity in the presence of A3G. HEK293T cells were co-transfected with an HIV-1 proviral plasmid pNL4-3 and pVR-A3G-cmyc for generation of the virions packaged with A3G in the presence of serially diluted peptides. Then, the culture supernatants containing the virions were collected at 48 h post-transfection for the viral infectivity assay using TZM-bl cells. The addition of VMP-60, VMP-63, and VMP-108 significantly reduced the infectivity of the A3G-containing viruses in a dose-dependent manner, and their IC_50_ were 97.0 μM, 49.4 μM, and 55.1 μM, respectively, while VMP-6, VMP-70, and VMP-111 did not affect the viral infectivity (Figure 2A). Since the inhibitory activity of VMP-63 was higher than that of VMP-60 and their sequences and mutation sites contained partial overlap, we chose VMP-63 and VMP-108 for the subsequent research. To confirm that the activity of the peptides was not a false-positive result due to cytotoxicity, we tested the cytotoxicity of VMP-63 and VMP-108 in HEK293T cells, which produced the virions. The results demonstrated that VMP-63 and VMP-108 had no significant effect on cell viability within the maximal working concentration (Figure 2B). Studies have shown that permissive cells, such as Jurkat cells, are more susceptible to HIV-1 replication than non-permissive cells, such as H9 cells, due to the absence of endogenous A3 expression [4,72]. To further verify that the antiviral activity of VMP-63 and VMP-108 is A3G-dependent, H9 and Jurkat cells that stably produce HIV-1 strain HXB2 under neomycin selection (H9/HXB2Neo and Jurkat/HXB2Neo) were used to test the antiviral effect of VMP-63 and VMP-108. The cells were incubated with different concentrations of the peptides for 48 h, and the culture supernatants containing the virions were collected to infect TZM-bl cells. The results showed that VMP-63 and VMP-108 significantly decreased the infectivity of virions produced from H9/HXB2Neo cells in a dose-dependent manner (Figure 2C,D). By contrast, none of them affected the infectivity of virions produced from Jurkat/HXB2Neo cells, indicating that the antiviral activity of VMP-63 and VMP-108 depended on intracellular A3G expression.

### 3.3. VMP-63 and VMP-108 Increase A3G Encapsulation into Viral Particles by Protecting A3G from Vif-Mediated Degradation

**Figure 3:**
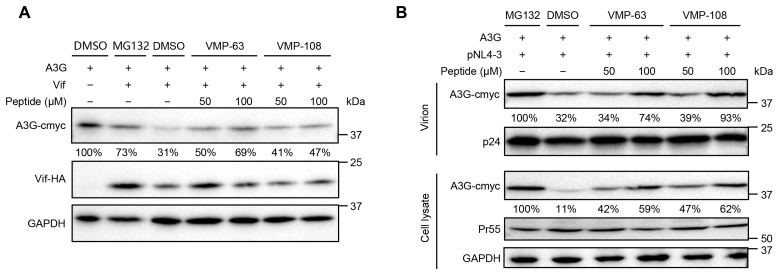
VMP-63 and VMP-108 rescued the level of A3G protein in transfected HEK293T cells and viral particles. (**A**) Detection of A3G levels in the presence of Vif with peptide treatment by Western Blotting. HEK293T cells were co-transfected with pVR-A3G-cmyc and pVR-Vif-HA and then incubated with 50 or 100 μM VMP-63 or VMP-108 or DMSO. A proteasome inhibitor, MG132, was used as a positive control. The expression level of A3G in the absence of Vif was set to 100%. The level of A3G was quantified by Image J and normalized with GAPDH. (**B**) The effect of peptides on the level of A3G packaged into viral particles. HEK293T cells were co-transfected with pVR-A3G-cmyc and pNL4-3 and then incubated with 50 or 100 μM of VMP-63 or VMP-108. After 48 h post-transfection, the cells and the supernatants containing viral particles were harvested for Western blotting. The levels of A3G in cell lysates or virions were normalized by the level of GAPDH or p24, respectively. The A3G level of the MG132 treatment group was set to 100%.

To confirm that the antiviral activity of VMP-63 and VMP-108 is achieved by protecting A3G, A3G levels in the presence of Vif were detected by Western blotting with or without the addition of VMP-63 and VMP-108. The presence of Vif reduced the expression level of A3G to 31% of that of the control group (Figure 3A). The addition of a proteasome inhibitor, MG132, rescued the level of A3G to 73%. By comparison, the addition of VMP-63 or VMP-108 also partially rescued the level of A3G. At a higher concentration of 100 μM, VMP-63 and VMP-108 increased the A3G level to 69% and 47%, respectively. To investigate whether VMP-63 and VMP-108 could enhance the encapsulation of A3G into nascent virions, HEK293T cells were transfected with pNL4-3 and A3G and incubated with the peptides for 48 h. We observed a significant recovery in the A3G levels in the cell lysates with the addition of 50 or 100 μM of VMP-63 and VMP-108, while in the viral particles only at the concentration of 100 μM of VMP-63 and VMP-108 (Figure 3B). These results indicate that VMP-63 and VMP-108 protect A3G from Vif-mediated degradation and thus enable more A3G to be packaged into nascent HIV-1 virions.

### 3.4. VMP-63 and VMP-108 Efficiently Enter Cells and Inhibit the Binding of Vif to CBFβ

**Figure 4:**
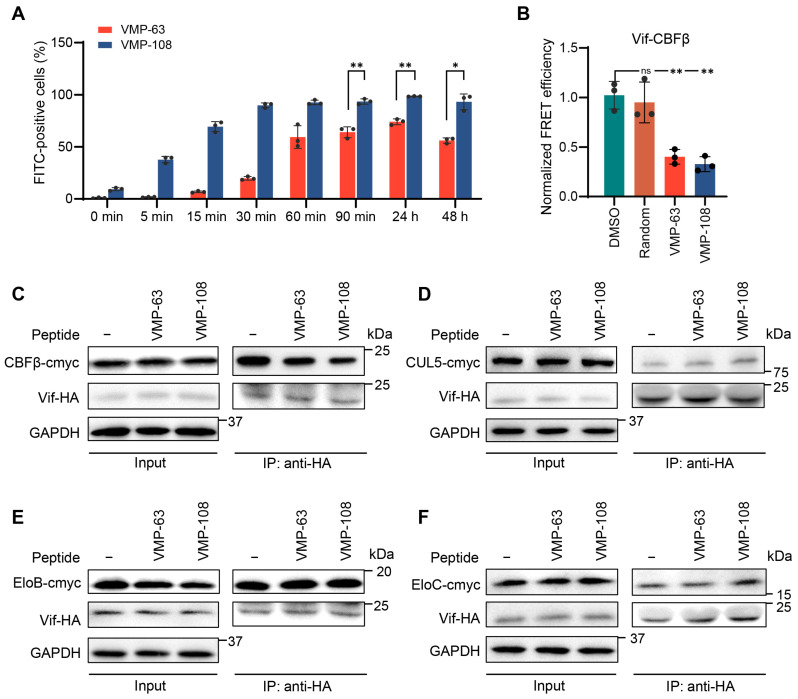
VMP-63 and VMP-108 could rapidly enter cells and inhibit the binding between Vif and CBFβ. (**A**) VMP-63 and VMP-108 could rapidly accumulate in cells. After the addition of 5 μM of FITC-labeled peptides to HEK293T cells, the percentage of FITC-positive cells increasing with time was analyzed by flow cytometry. The mean ± SD values of three independent experiments are shown. (**B**) VMP-63 and VMP-108 blocked the Vif-CBFβ interaction detected by fluorescence resonance energy transfer (FRET). HEK293T cells were transfected with Vif-YFP and CBFβ-CFP and then incubated with 100 μM VMP-63 or VMP-108, random peptide, or DMSO for 48 h. The peptides significantly reduced the FRET efficiency between Vif and CBFβ. The normalized FRET efficiency is calculated by the E (FRET) values from Table 3. The average FRET efficiency of the DMSO-treated group was set to 1.0. Statistical significance was assessed by the Student’s *t*-test. (**C**–**F**) The peptides specifically affected the interaction of Vif with CBFβ in the E3 complex. HEK293T cells were co-transfected with Vif-HA and CBFβ-cmyc (**C**), CUL5-cmyc (**D**), EloB-cmyc (**E**), or EloC-cmyc (**F**) with or without the addition of VMP-63 or VMP-108. After 48 h of transfection, the cells were harvested and analyzed by Co-IP experiments. * indicates *p* < 0.05; **, *p* < 0.01; ns, not significant.

To investigate whether the two peptides can efficiently enter cells to act on the intracellular target, we synthesized FITC-labeled VMP-63 and VMP-108 and detected the percentage of FITC-positive cells with time by flow cytometry, immediately after the addition of the peptides to HEK293T cells. The results showed that about 70% of cells had taken in VMP-63 within 90 min, while this percentage for VMP-108 quickly occurred within 15 min (Figure 4A). After 30 min of incubation, more than 90% of cells contained VMP-108. After 24 h, the percentages of VMP-63- and VMP-108-positive cells reached the top levels of 74.1% and 98.5%, respectively, and then decreased. However, more than 90% of cells were still positive at 48 h post addition of VMP-108 (Figure 4A), suggesting that VMP-108 accumulates quickly and more in the cells and is more stable than VMP-63. To verify whether the peptides could competitively bind to CBFβ instead of Vif, subsequently protecting A3G from degradation, we analyzed the inhibition effects of VMP-63 and VMP-108 on the interaction between Vif and CBFβ by a FRET assay. Vif-YFP and CBFβ-CFP were co-transfected into HEK293T cells with the addition of 100 μM VMP-63 or VMP-108. Compared with the DMSO-treated group, the presence of VMP-63 or VMP-108 significantly reduced the FRET efficiency represented by the E (FRET) values in three independent replicates, while the random peptide did not show the effect (Table 3 and Figure 4B), indicating that the peptides blocked the interaction between Vif and CBFβ. To analyze whether the addition of peptides affects the interactions between Vif and the other proteins in the E3 complex, we co-transfected Vif-HA with CBFβ, CUL5, EloB, or EloC into HEK293T cells with or without the addition of VMP-63 or VMP-108, and the content changes of Vif-bound proteins were analyzed by Co-IP. The results showed that the addition of VMP-63 and VMP-108 significantly reduced the interaction between Vif and CBFβ but had no effect on Vif binding to CUL5, EloB, and EloC, suggesting the specific inhibition of Vif-CBFβ interaction by the peptides (Figure 4C–F).

**Figure 5:**
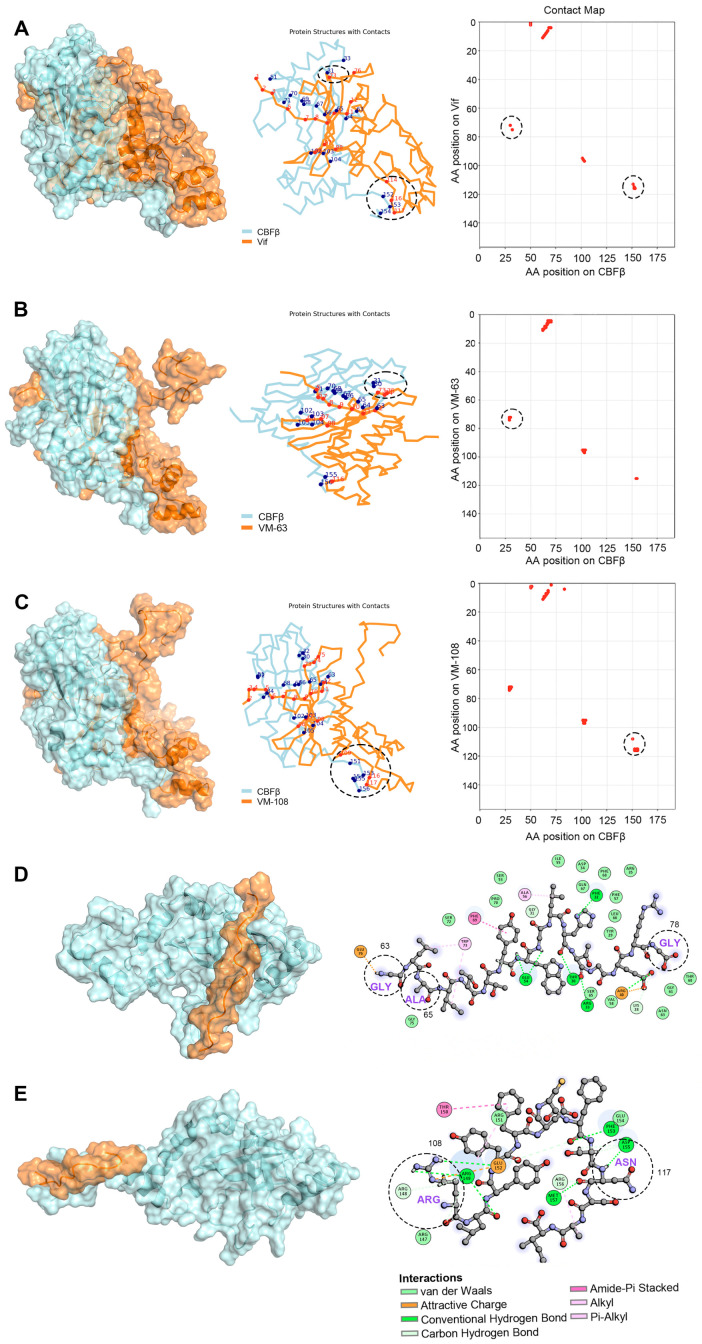
Representative conformations of Vif and the Vif mutants with CBFβ. (**A**–**C**) Depiction of the CA backbone (left), interacting residues (middle), and contact matrices (right) for the interaction between CBFβ and WT Vif (**A**), a Vif mutant with the mutations in region 63 to 78 as VMP-63 (VM-63) (**B**), or a Vif mutant with the mutations in region 108 to 120 as VMP-108 (VM-108) (**C**). The interacting residues on Vif and CBFβ were indicated in red and blue dots, respectively. The position and number of the interacting sites in the regions 63 to 78 and 108 to 120 of Vif were marked in dashed circles. (**D**,**E**) Illustration of the representative conformations of VMP-63 (**D**) and VMP-108 (**E**) peptide-CBFβ complexes. The interacting sites on CBFβ (indicated in colored circles) with the peptides and the interaction forces are highlighted on the right. The mutated residues in the peptides are indicated in dashed circles.

Subsequently, to confirm whether the Vif protein with the mutations in the AA regions from 63 to 78 (VM-63) or 108 to 120 (VM-108) corresponding to VMP-63 or VMP-108, respectively, has a binding advantage with CBFβ over WT Vif, we performed MD simulation studies based on the Vif-CBFβ structure derived from the crystal structure of the E3 complex (PDB: 4N9F) [37]. After MD simulation, K-means clustering based on the positions of heavy atoms was employed to analyze the representative conformations of each system (Figure 5A–C). The CA coordinates from the PDB were extracted, and a cutoff distance of 6 Å was set for residue contacts between the two proteins. In the AA regions from 63 to 78 and 108 to 120, the wild-type Vif protein was in contact with the corresponding amino acids of CBFβ at positions 73, 114, 116, and 117, respectively (Figure 5A). The mutations at R63G and V65A changed the binding conformation between Vif and CBFβ, resulting in an increase in the contact area of VM-63 with CBFβ at positions 73, 74, and 75 (Figure 5B). The two mutations on VM-108, H108R, and D117N also promoted the interaction of Vif at positions 109, 116, and 117 with CBFβ, increasing the number of contact sites of CBFβ from 3 to 5 (Figure 5C). The MM-PBSA results illustrated the differences in binding energies between WT Vif and the mutants with CBFβ (Table 4). Both of the mutants showed improved binding efficacy compared to WT Vif, with energies of −172.29 and −166.41 kcal/mol, respectively, which supports our initial screening results of Vif mutants with higher affinity to CBFβ. Notably, despite the CBFβ-VM-108 complex exhibited higher van der Waals and electrostatic forces (ΔE_vdW_ and ΔE_ele_), its higher solvation energy (ΔG_solv_) led to a similar overall ΔG_total_ with the CBFβ-VM-63 complex, which is a characteristic of the MM-PBSA calculation mechanism. Further analysis of the peptide-CBFβ complexes using K-means clustering revealed differences in the binding sites of the peptides with CBFβ compared to the complete Vif protein (Figure 5D,E). Notably, VMP-108M induced significant conformational changes in CBFβ, potentially affecting the normal interaction of CBFβ with Vif and suggesting a correlation with its enhanced inhibitory properties.

### 3.5. VMP-108 Inhibits HIV-1 Replication in A3-Positive T Lymphocytes

**Figure 6:**
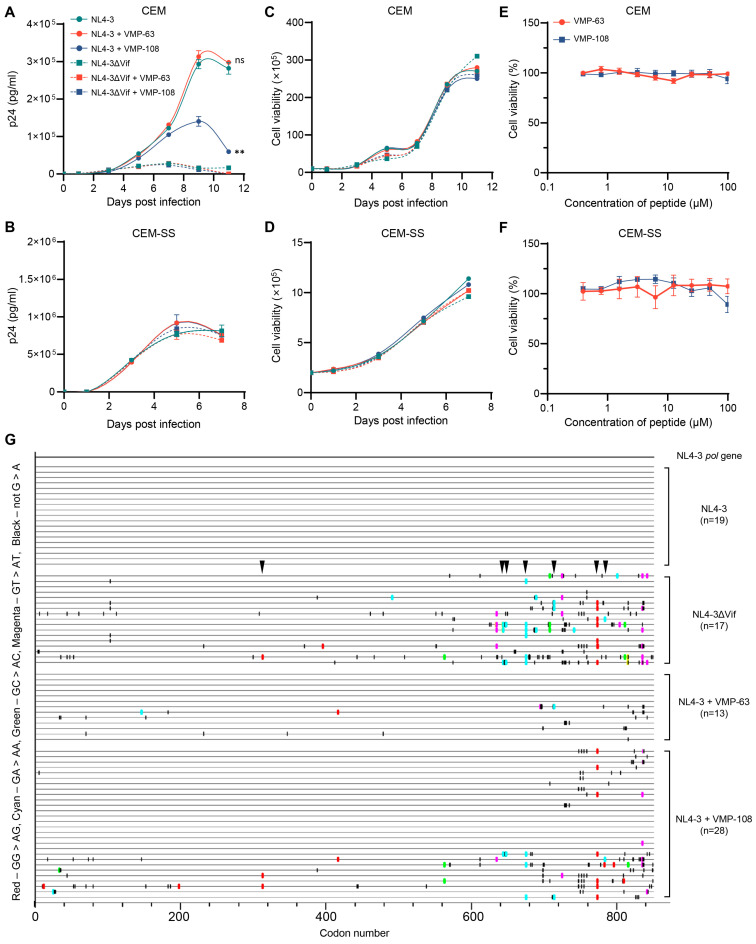
VMP-108 specifically inhibited the replication of HIV-1 in nonpermissive T cells. (**A**,**B**) VMP-108 restricted the replication of NL4-3 in CEM cells. NL4-3 (solid circles) and NL4-3ΔVif (solid squares) of equal amounts of p24 were used to infect CEM (**A**) and CEM-SS (**B**) cells, and the viral production was monitored for 11 or 7 days and quantified every other day using a p24 ELISA kit. The peptides were added at 50 μM and replenished every 48 h. (**C**,**D**) The cell viability of the infected cells. The CEM (**C**) and CEM-SS (**D**) cells infected above were quantified by trypan blue staining every other day. (**E**,**F**) Different concentrations of VMP-63 and VMP-108 showed no cytotoxicity on CEM (**E**) and CEM-SS (**F**) cells. The peptides were diluted by 2-fold from 100 μM and incubated with the cells for 48 h. The cell viability of the DMSO-treated group was set to 100%. Error bars represent the SD calculated from three independent infections. Statistical significance was assessed using a two-way ANOVA between the indicated and the positive control curves. (**G**) Highlighter plot analysis of the *pol* gene sequences. The *pol* gene fragments (about 850 bp) from the culture supernatant samples collected on day 11 from the infected CEM cells of (**A**) were amplified to analyze the frequency of A3-induced G-to-A mutations (labeled in red and cyan). Red, GG-to-AG mutations. Cyan, GA-to-AA mutations. Green, GC-to-AC mutations. Magenta, GT-to-AT mutations. Black, non-G-to-A mutations. The locations indicated by black arrows show the same A3-induced G-to-A mutation sites that occurred in the NL4-3ΔVif and NL4-3+VMP-108 groups.

To investigate whether VMP-63 and VMP-108 could inhibit the long-term viral replication, CEM and CEM-SS cells, which are non-permissive and permissive T lymphocytes, respectively, were infected with HIV-1 NL4-3 or NL4-3ΔVif of equal amounts of p24 and incubated with 50 μM peptides for 11 or 7 days, depending on the viral replication rate. The results showed that VMP-108 significantly inhibited the replication of NL4-3 in CEM cells (Figure 6A). However, VMP-63 did not affect the replication curve of NL4-3. Due to the absence of Vif to counteract the endogenous A3 proteins, viral replication was not restricted by the peptides in the NL4-3ΔVif-infected CEM cells. The peptides also did not impact the replication curves of NL4-3 and NL4-3ΔVif in CEM-SS cells, which have no endogenous A3 expression (Figure 6B). Meanwhile, the growth of CEM and CEM-SS cells was not affected by the addition of VMP-63 or VMP-108 (Figure 6C,D). We also tested the cytotoxicity of the peptides at different working concentrations. VMP-63 and VMP-108 demonstrated almost no cytotoxicity on the two cell lines (Figure 6E,F).

To confirm that the VMP-108-induced inhibition of viral replication was achieved by the protection of A3 function, we amplified part of the *pol* gene from the last time point samples of the viral supernatants of the NL4-3, NL4-3ΔVif, NL4-3+VMP-63, and NL4-3+VMP-108 groups collected from the infected CEM cells. By alignment of the amplified sequences, none of the mutations were found in the 19 sequences derived from the NL4-3 group in the *pol* gene region (Figure 6G). In contrast, in the 17 sequences derived from the NL4-3ΔVif group, 13 sequences (76.5%) contained A3-induced G-to-A mutations due to the higher levels of endogenous A3 proteins. In the 13 sequences, 9 GG-to-AG mutations on 3 independent sites and 19 GA-to-AA mutations on 9 independent sites were observed, an average of 2.15 G-to-A mutations per sequence. Only 2 of the 13 sequences (15.4%) derived from the VMP-63 treatment group had A3-induced G-to-A mutations, while 11 of the 28 sequences (39.3%) from the VMP-108 treatment group contained the mutations. In the 11 sequences with A3-induced G-to-A mutations, 15 GG-to-AG mutations on 9 independent sites and 8 GA-to-AA mutations on 6 independent sites were observed, averaging 2.09 G-to-A mutations per sequence, and 7 mutation sites were also observed in the sequences from the NL4-3ΔVif group. We also found that with the increase in G-to-A mutation frequency, the frequency of non-G-to-A mutations in this region also increased. These results were consistent with the viral replication levels detected in these groups, indicating that VMP-108 significantly inhibited long-term HIV-1 replication in T lymphocytes by rescuing the endogenous levels of A3 proteins.

## 4. Discussion

Compared with other types of anti-HIV drugs, peptide inhibitors have the advantage of selectivity and specificity towards the targets with lower side effects and toxicity, and can be used in combination with other anti-HIV drugs. Existing anti-HIV peptide inhibitors mainly target the fusion of the virus with the host cell membrane and the catalytic site of viral integrase. Enfuvirtide (T20) is the only FDA-approved anti-HIV fusion inhibitor, which functions by binding to the viral membrane protein gp41 [73]. Later studies improved the activity of T20 by increasing its half-life and reducing the IC_50_ value to the picomole level [74]. In addition, peptides derived from the sequence of the integrase-binding domain (IBD) of lens epithelial-derived growth factor (LEDGF) have been shown to inhibit the catalytic function of HIV-1 integrase by altering the oligomerization of integrase [75,76]. Peptides derived from HIV-1 Vpr also exhibit a micromolar level of inhibition of integrase catalysis in in vitro experiments [77].

Existing studies of Vif-derived peptide antagonists targeting the Vif-A3G-E3 complex have tested the Vif-Vif and Vif-A3G interactions as targets [47,48,49,50]. Here, we develop peptide inhibitors targeting the Vif-CBFβ interaction, which has not been tested for the probability of peptide blocking before. Our study demonstrates that VMP-63 and VMP-108 specifically reduce the infectivity of HIV-1 produced from A3G-positive cells by directly inhibiting the interaction between Vif and CBFβ. The recovery of intracellular A3G levels from degradation indicates that the binding of the peptides to CBFβ impairs the ability of Vif to recruit the E3 ubiquitin ligase complex (Figure 3 and Figure 4B,C). Therefore, the proportion of A3G packaged into viral particles was also significantly increased, subsequently leading to the antiviral outcomes. Compared to a small-molecule inhibitor CV-3 targeting the same interaction, which was identified in our previous study [44], VMP-108 and VMP-63 inhibited 31.7% and 26.7% of infections at a concentration of 50 μM in chronically infected H9/HXB2Neo cells, respectively (Figure 2C,D), while CV-3 at the same concentration inhibited almost 100% of infections in the same cell line (IC_50_ = 8.16 μM). Although the magnitude of the IC_50_ of the newly developed peptides is higher than CV-3, they showed lower cytotoxicity in T cell lines such as CEM, which broadens their scale of usage. Further investigation needs to be conducted to improve the activity of VMP-63 and VMP-108 at lower concentrations by shortening the peptides to the core functional regions and applying modifications such as PEGylation, lipidation, and D-amino acid substitutions [78].

Yeast surface display technology is an effective method for screening inhibitors targeting PPIs [79], with advantages in the expression of proteins or peptides that are difficult to fold and secrete and achieving high-throughput screening [80]. Although displaying randomized peptides on the surface of yeast cells allows direct screening of Vif peptides with higher affinity to CBFβ, our peptide design strategy based on the identified high-affinity Vif mutants is more rational by taking both functional domains and mutations into consideration. By using Vif mutation plasmid libraries with high pool capacity, random mutations could occur at any site in the Vif sequence. In our previous study, 50 sequences of Vif mutants with higher affinity for CBFβ were obtained by yeast surface displaying of Vif mutant libraries [45]. Similarly, 29 new Vif mutant sequences were obtained herein. More than 90% of the mutation sites in the new sequences are consistent with our previous results, and the MD simulation studies demonstrated that the Vif mutants containing the screened mutations had a binding advantage with CBFβ over WT Vif (Figure 5A–C), which proves the stability and feasibility of this screening platform. In addition to the mutation sites Q6E/L, V7E, W11G, I87V, A103P, D104N, and H108R, which are reported to directly impact the interaction with CBFβ [36,39,41,43], mutations R19T, K22E, V25A, R63G, V65A, and E76K also appeared at a high frequency in all screened mutant sequences.

The design of peptides was mainly based on the three key interacting regions of Vif with CBFβ [37]. VMP-6 contains the key regions of ^5^WQVMIVW^11^ and R19T. VMP-60, VMP-63, and VMP-70 contain R63G, V65A, E76K, and the two key sites L64 and I66. VMP-108 and VMP-111 contain F112, F115, and the key site H108 of the zinc finger domain. Although the dominant-negative mutant Vif-6M only carrying K22E and V25A double mutations showed potent antiviral effects by competitively inhibiting the Vif-CBFβ interaction in our previous study [45], these two sites are not located in the structural interacting domains of Vif with CBFβ. Therefore, the sequence of Vif-6M does not conform to the peptide design criteria herein. Among the peptides, only VMP-60, VMP-63, and VMP-108 effectively reduced the infectivity of the virus (Figure 2A). VMP-63 contains the entire sequence of VMP-70, but VMP-70 showed no inhibition activity, indicating that the ^63^GLAITTY^69^ region contributes to the inhibitory effect of VMP-63 (Table 2). Compared with VMP-60, which also shares the same functional region, VMP-63 showed a lower IC_50_ against HIV-1, suggesting that the R63G mutation is crucial to the antiviral activity of the functional region, and the V65A mutation between the two key sites L64 and I66 further improves the activity. Consistently, the MD simulation studies demonstrated that the R63G mutation of VMP-63 interacts with residue 76 of CBFβ through an attractive charge force (Figure 5D). Meanwhile, the V65A mutation in VMP-63 changes the angle between L64 and I66 on the peptide, resulting in a van der Waals force with the residue 75 of CBFβ and a Pi-Alkyl interaction with the residue 73 of CBFβ (Figure 5D). VMP-108 and VMP-111 also have an overlapping region from Y111 to A119 in the sequences, but their antiviral effects are completely opposite, indicating that the key mutation H108R contained by VMP-108 largely determines the antiviral activity. In the MD simulation studies, the H108R mutation of VMP-108 interacts with CBFβ through an attractive charge force and hydrogen bond (Figure 5E). In contrast, the WT Vif protein has no interactions with CBFβ at these locations (Figure 5A). The results of the MD simulation studies also showed more residues of CBFβ interacting with the peptides than with the WT Vif protein (Figure 5D,E), suggesting a competitive role of the peptides in blocking the binding of Vif to CBFβ.

Interestingly, we found that VMP-63 did not effectively restrict the long-term replication of HIV-1 (Figure 6A), although it demonstrated equivalent ability to VMP-108 in protecting A3G (Figure 3) and competing with Vif to bind CBFβ (Figure 4B,C). The cell uptake rate of peptides that act on intracellular targets is a limiting factor of the inhibition activity and may influence the actual IC_50_ value. VMP-108 could rapidly enter cells and accumulate more in cells than VMP-63, as we observed (Figure 4A). In addition, VMP-108 maintained a much higher level in cells than VMP-63 after 48 h of incubation, suggesting that it may have a higher actual working concentration in cells over a longer duration. These advantages may lead to the better performance of VMP-108 in inhibiting the long-term replication of HIV-1.

Together, by identifying VMP-108, which efficiently restricts the infection and long-term replication of HIV-1, the present study provides a novel strategy for anti-HIV peptide design based on high-affinity dominant-negative mutants and potential peptide candidates for acquired immune deficiency syndrome therapy.

## Figures and Tables

**Table 1:** Summary of Vif mutation information.

Vif Mutation Site	Functional Region
Q6E, Q6L, V7E, Q12E, R19T *	CBFβ binding domain [41,43]
K22E, V25A, M29V, E45K	APOBEC3G binding domain [66]
G60R, R63G, V65A	CBFβ binding domain [36]
E76K *, D78G	APOBEC3F binding domain [66]
I87V	APOBEC3G/CBFβ binding domain [39,40]
E101G, A103P, D104N *	CBFβ binding domain [39]
H108R, D117N/H, I120T, A123V	CBFβ/Cullin5 binding sites [39,42,69,71]
A151P	–
PP161/162AA	APOBEC3G/Elongin B binding sites [66,70]
S165G *, T167K, K168N, R173T, Q178H *, G182V	APOBEC3F binding domain [66]

* Mutation sites occur more than twice in the sequences of Vif mutants. The sites marked with underlines are amino acids that have been reported to be critical to Vif binding with CBFβ [36,39,41,43].

**Table 2:** The characteristics of peptides designed.

Peptide Name	AA Region	Sequence	Length (AA)	Solvent
VMP-6	6–20	EVMIVWEVDRMRITT	15	DMSO
VMP-60	60–76	RDAGLVITTYWGLHTGK	17	DMSO
VMP-63	63–78	GLAITTYWGLHTGERG	16	DMSO
VMP-70	70–78	WGLHTGERG	9	H_2_O
VMP-108	108–120	RLYYFDCFSNSAI	13	DMSO
VMP-111	111–123	YFDCFSNSATRKV	13	DMSO

The mutation sites contained in the peptides were marked with underlines. AA, amino acid.

**Table 3:** The FRET efficiency between Vif and CBFβ inhibited by the peptides.

Group	E (FRET) *-1	E (FRET)-2	E (FRET)-3	Mean ± SD
DMSO	21.21	16.21	20.04	18.71 ± 2.5
Random peptide	14.78	21.1	14.73	17.92 ± 3.18
VMP-63	10.16	5.37	7.18	7.76 ± 2.4
VMP-108	8.71	4.28	4.65	6.49 ± 2.2

* E (FRET) = FRET/F510 × 100. In the formula, the value of FRET is the maximum fluorescence intensity at 530 nm in the FRET spectrum, and the value of F510 is the maximum fluorescence intensity of the YFP sample in the corresponding group at 530 nm after excitation at 510 nm. The E (FRET) values were calculated from three independent experiments.

**Table 4:** MM-PBSA results of binding energies of the complexes, expressed in kcal/mol.

System (kcal/mol)	CBFβ-Vif	CBFβ-VM-63	CBFβ-VM-108
ΔE_vdW_	−189.85	−272.89	−309.32
ΔE_ele_	−607.6	−928.14	−1063.52
ΔG_gas_	−797.01	−1201.03	−1372.84
ΔG_solv_	682.27	1028.74	1206.43
ΔG_total_ *	−114.74	−172.29	−166.41

* ΔE_vdW_ is the nonbonding van der Waals energy. ΔE_ele_ is the nonbonding electrostatic energy. ΔG_gas_ = ΔE_vdW_ + ΔE_ele_. ΔG_solv_ is the solvation energy. ΔG_total_ = ΔG_gas_ + ΔG_solv_.

## Data Availability

Data are contained within the article.
